# Crowdsourced RNA design discovers diverse, reversible, efficient, self-contained molecular switches

**DOI:** 10.1073/pnas.2112979119

**Published:** 2022-04-26

**Authors:** Johan O. L. Andreasson, Michael R. Gotrik, Michelle J. Wu, Hannah K. Wayment-Steele, Wipapat Kladwang, Fernando Portela, Roger Wellington-Oguri, Eterna Participants, Rhiju Das, William J. Greenleaf

**Affiliations:** ^a^Department of Genetics, Stanford University School of Medicine, Stanford University, Stanford, CA 94305;; ^b^Department of Biochemistry, Stanford University School of Medicine, Stanford University, Stanford, CA 94305;; ^c^Biomedical Informatics Training Program, Stanford University School of Medicine, Stanford University, Stanford, CA 94305;; ^d^Department of Chemistry, Stanford University, Stanford, CA 94305;; ^e^Eterna Massive Open Laboratory;; ^f^Department of Physics, Stanford University, Stanford, CA 94305;; ^g^Department of Applied Physics, Stanford University, Stanford, CA 94305;; ^h^Chan-Zuckerberg Biohub, San Francisco, CA

**Keywords:** RNA, crowdsourcing, RNA sensor, high throughput, design

## Abstract

Internet-based scientific communities promise a means to apply distributed, diverse human intelligence toward previously intractable scientific problems. However, current implementations have not allowed communities to propose experiments to test all emerging hypotheses at scale or to modify hypotheses in response to experiments. We report high-throughput methods for molecular characterization of nucleic acids that enable the large-scale video game–based crowdsourcing of RNA sensor design, followed by high-throughput functional characterization. Iterative design testing of thousands of crowdsourced RNA sensor designs produced near–thermodynamically optimal and reversible RNA switches that act as self-contained molecular sensors and couple five distinct small molecule inputs to three distinct protein binding and fluorogenic outputs. This work suggests a paradigm for widely distributed experimental bioscience.

## Significance

Our manuscript presents a paradigm for carrying out distributed science. We have harnessed an online RNA design game, Eterna, to challenge a large community of RNA designers to create diverse RNA sensors. RNA is an attractive, biocompatible substrate for the design and implementation of molecular sensors. We tasked the diverse Eterna community, comprising a global network of molecular design enthusiasts, to submit thousands to tens of thousands of “solutions” to these RNA sensor design challenges. Crucially, community designs were synthesized and tested experimentally in the real world using high-throughput methods for biochemical assays built on repurposed DNA sequencers. The best player-generated designs for RNA sensors approached the thermodynamic optimum.

Several recent platforms have demonstrated the engagement, via the internet, of vast untapped human cognitive potential, transforming the scale by which scientific questions can be approached with human intelligence. However, previous work has only allowed “citizen scientists” to process data already collected (1, 2), to interface with purely computational methods to implement solution strategies (3), or to compete for limited slots on a low-throughput experimental synthesis pipeline (4–6). We reasoned that by enabling a community of RNA design enthusiasts to both generate hypotheses and then acquire large-scale experimental evidence supporting or rejecting these hypotheses, we could “close the loop” on crowdsourced science, providing a platform for iterative hypothesis generation and experimental testing. Recent advances in high-throughput functional characterization of nucleic acids on sequencing instruments provide potentially immense capacity for carrying out experiments devised by these scientific communities (7–9). We hypothesized that with these tools, online communities would be capable of producing RNA sensors with unprecedented performance.

RNA is an attractive substrate for diagnostics (10), cellular computing (11), “smart” imaging tools (12), and targeted therapeutics (13, 14) due to its inherent biocompatibility (with sensors often transcribed from DNA due to the lability of RNA itself), capacity for large-scale structural rearrangements via alternative base pairings, and ability to form aptamers that specifically recognize cellular metabolites, signaling molecules, ions, cofactors, and proteins (15, 16). Riboswitches, a widely biologically utilized form of RNA sensor, generally sense biomolecules and respond by causing RNA cleavage (17), transcription termination (18), or other altered genetic outputs. We will refer to self-contained (i.e., those that do not rely on the mechanics of the transcription process) and reversible (i.e., those that do not rely on irreversible processes) switches as “stand-alone” RNA switches. Such stand-alone RNA switches can be applied to important applications, such as fluorescence-based sensors of rapid, dynamic processes or reversible chemical control of therapeutics (19). To date, all stand-alone RNA switches have exhibited less than 10-fold changes in output signal in the presence vs. absence of ligand—far below the limits allowed by thermodynamics (20)—and a thermodynamically reversible RNA switch has not been demonstrated. Thus, a method to design efficient, stand-alone RNAs capable of conformationally responding to inputs both sensitively and reversibly is needed. This challenge in RNA design provides an ideal test bed for distributed, internet-based science.

**Fig. 1.**
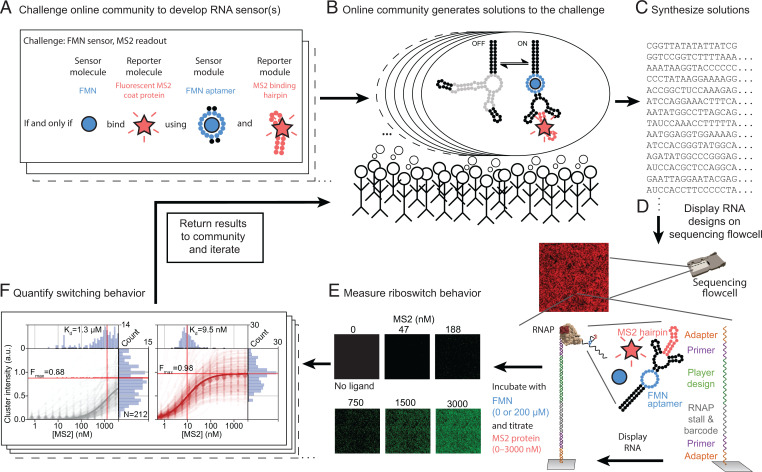
A platform for crowdsourced RNA sensor design and testing at high throughput. (*A*) RNA switch sensor design challenges are released to the Eterna community. In this example, an FMN aptamer (small blue circles) should be used to sense the FMN molecule (large blue circles), and in the presence of FMN, an MS2 hairpin (red circles) must fold for detection via binding of the fluorescent MS2 coat protein (red stars). (*B*) Eterna community designs sequences of RNA switch predicted to fold into appropriate off and on states. (*C*) Puzzle solutions are synthesized by DNA array synthesis and converted to libraries ready for RNA-MaP via PCR. (*D*) Following high-throughput sequencing of the library on an Illumina sequencing chip, a biotinylated primer is annealed and extended to create double stranded DNA. Streptavidin is then bound, and RNA polymerase (RNAP) displays one RNA switch variant solution per sequencing cluster. (*E*) Binding of the fluorescently labeled MS2 coat protein is then quantified across all clusters at increasing concentrations (example images are shown). Cluster sequence information is used to link fluorescent signals to underlying RNA switch sequence. (*F*) Binding data are quantified from multiple clusters for a single RNA switch variant (median fits are shown as the bold line, a.u. are arbitrary units). These data are then released to the Eterna community, and subsequent rounds of designs are solicited.

To implement a platform for hypothesis-based, iterative scientific discovery for RNA sensors, we combined internet-scale crowdsourcing of RNA design using the scientific discovery game Eterna (5) and high-throughput, quantitative RNA characterization using repurposed sequencing chips and instruments (7, 8). Eterna puzzles challenge an online player community to design RNA sequences that couple the formation of a ligand-binding input element (Fig. 1*A*) to structure changes that promote or prevent the formation of a second sequence-defined output element. These output elements provide measurable fluorescent readouts proportional to a switch’s response. The Eterna platform enables players to design sequences that will fold differentially in the presence of ligand using standard thermodynamic modeling packages to estimate free energies of RNA folding and to simulate binding of the ligand. While providing rapid computational feedback, these packages provide imperfect approximations of the expected folding of model designs (21), necessitating both human insight and experimental testing. Importantly, we provided no constraints on the tools that individuals could use when generating their solutions, allowing any algorithmic methods (including Ribologic, described below) to be incorporated. This “anything goes” approach to design allowed designers to use the combination of both human intuition and algorithmic exploration. We solicited tens of thousands of Eterna designs from the online community (Fig. 1*B*) and then characterized these designs using massively parallel RNA array technology. Briefly, player designs were displayed on Illumina sequencing chips, and switch behavior was evaluated under relevant conditions (RNA-MaP [RNA on a massively parallel array]) (Fig. 1 *C–E*). This platform quantifies the switch function by measuring binding curves for every design interacting with a fluorescent reporter molecule in both the presence and the absence of the input ligand (Fig. 1*F*) and achieves excellent reproducibility (*R*^2^ = 0.94) (*SI Appendix*, Fig. S1) (21–23). Results from these experiments were then released to the Eterna community, and further design refinement was solicited (Fig. 1*A*). In this study, we define the activation ratio (*AR*) of the RNA switch as the fold change in the observed *K_D,obs_* between the off state (weak reporter ligand binding) and the on state (strong reporter ligand binding). In the limit of low reporter (output ligand) concentrations, this fold change in *K_D,obs_* is equal to the ratio of measured intensities commonly reported for switches in literature. In contrast to measuring signals at single reporter concentrations, however, we determined changes in the signal from the full range of on-state reporter affinities, which allows for maximized absolute signal changes and for quantities to be directly related to biophysical models (20).

**Fig. 2.**
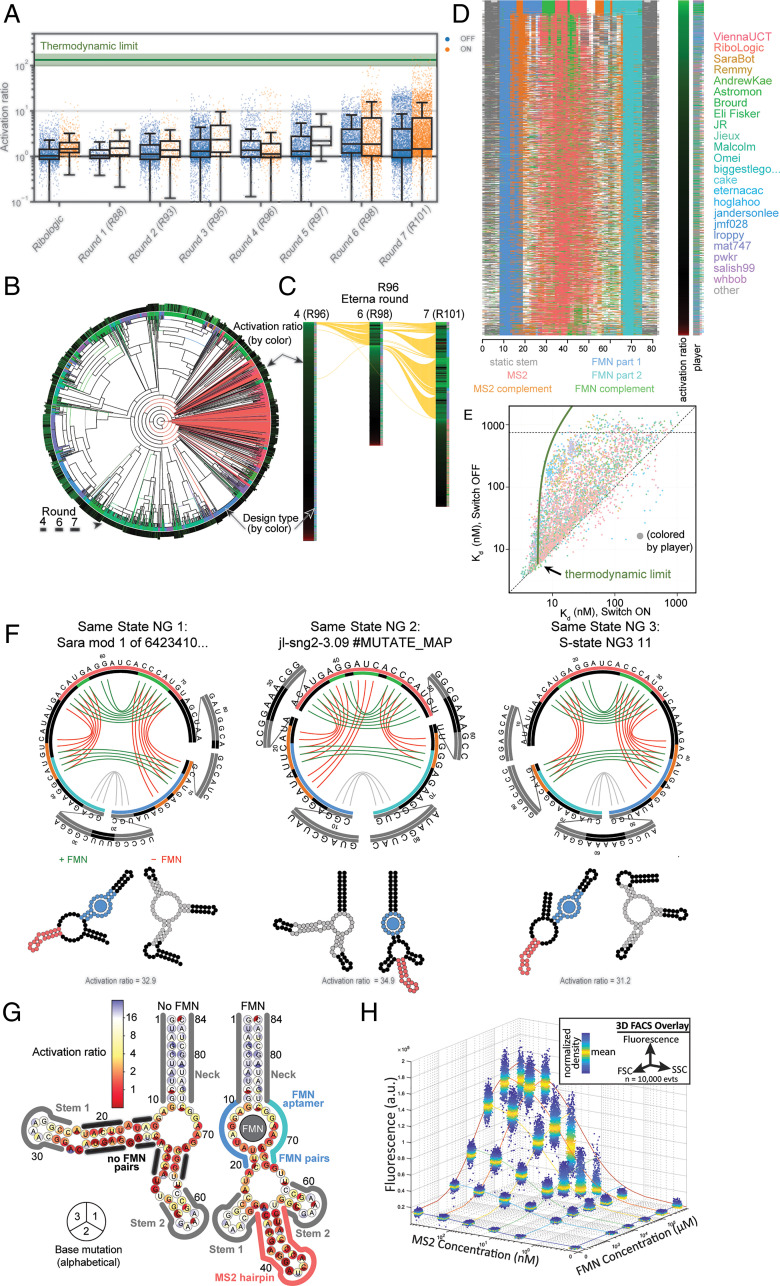
Performance of player-designed sensors. (*A*) Box plots (interquartile range [IQR] with whiskers at ±1.5 IQR) for the *AR* for on (blue points) and off (orange points) FMN–MS2 switches over seven rounds show substantial improvement as players learn from prior rounds of design and outperform automated Ribologic design. The theoretical thermodynamic limit for the *AR*, estimated using the measured intrinsic *K*_d_ of the FMN aptamer, is depicted as a green line. (*B*) Phylogenetic tree of puzzle solutions for an FMN–MS2 on switch (“same-state NG 2”). Terminal branches are colored by design type: player designs (green), automated solutions from Ribologic (red), and modifications of previous designs (blue). The outer ring is colored by the *AR* (brighter green is higher, red is less than one), and the width represents the round of submission (one to three). (*C*) Same-state NG 2 solutions by round color coded as in *B*. Yellow lines connect “mods,” defined as sequences separated by Levenshtein edit distance less than five. (*D*) Position of functional elements across all same-state NG 2 solutions color coded as indicated and sorted by *AR*. The MS2 and FMN complements are defined as parts of the sequence predicted to pair with any part of the MS2 hairpin or FMN aptamer, respectively. (*E*) *K*_d_ values for both states of the same-state NG 2 puzzle designs. The predicted thermodynamic limit for the *K*_d_ in the off state, based on the *K*_d_ in the on state, is shown in green. (*F*) Predicted secondary structures for the best solutions from three on-switch puzzles with predicted invariant base pairs (gray segments) and base pairs in the absence (red) or presence (green) of the FMN ligand. Outer circles display the MS2 hairpin and complementary segments in color; inner circles display the FMN aptamer and complementary segments. (*G*) *AR* for switching by mutants of the top-scoring design mapped by color onto predicted secondary structures. Blue implies less disruption of switching upon mutation. Perturbations of single mutations for each base are indicated by color within circle sectors (A, C, G, or U proceeding clockwise from the top and omitting the original base). Sequence segments representing functional (FMN in blue/turquoise, MS2 in red), invariant (gray), or other secondary structures elements are outlined by color. (*H*) Fluorescence intensity of particle display beads under varying concentrations of FMN and MS2 measured by flow cytometry (FACS). Each cluster of points is a three-dimensional overlay of a scatterplot showing the measured forward scatter (FSC), side scatter (SSC), and fluorescence intensity (a.u. are arbitrary units) of an RNA-coated particle centered at a given FMN and MS2 concentration. Colored lines represent the global fit of the mean fluorescent values at each concentration to an equilibrium binding model.

As a first test, we challenged players to couple input and output RNA aptamers that were individually well characterized but had not been previously combined into an RNA switch: an 11-nt aptamer for the cellular metabolite flavin mononucleotide (FMN; input) (24) and a 19-nt hairpin that binds the bacteriophage MS2 coat protein (output) (7). The maximum activation ratio (*AR*_max_) expected for this challenge, as determined by the dissociation constant of the FMN aptamer and the experimental trigger concentration of input ligand, was 132 ± 33 (20) (*SI Appendix*, *Materials and Methods*). Over seven consecutive rounds of design, synthesis, characterization, and community-wide discussion, 149 members of the Eterna community submitted over 30,000 designs (*SI Appendix*, Fig. S1 and Table S4). Of these, 30 designs from five distinct individuals fell within error of the thermodynamic maximum (Fig. 2*A* and *SI Appendix*, Fig. S2 and Table S2).

In community-wide discussions, players attributed their success to two primary factors: 1) the relaxation of puzzle design constraints (enabled through updates of the Eterna source code) that allowed for exploration of a much larger design space and 2) the public dissemination of annotated experimental data that let players iterate and improve upon any past design. Designs exhibited striking improvements over iterative rounds; in the first round, the *AR*_max_ value achieved was 5.7, while by the last rounds, several designs exhibited *AR* values in excess of 80. The most dramatic improvement occurred after the fourth round when constraints on the ordering of the FMN and MS2 hairpin were eliminated (*SI Appendix*, Fig. S1 and Table S1). This game update allowed the community to autonomously explore a wide variety of such element orderings (i.e., tandem or split-aptamer arrangements) in remaining rounds (represented as clades in Fig. 2*B*).

Changes to design constraints and iterative refinement of promising solutions (Fig. 2*C*) yielded a collection of structural mechanisms (i.e., orderings of aptamer elements and design of sites reverse complementary to those elements) (Fig. 2*D*) that reliably yielded high *AR* values, often approaching the thermodynamic *AR*_max_ of 132 (the green bar in Fig. 2*A*). As expected from theoretical predictions (20), these top designs exchange lower output levels in the activated (reporter-bound) state in exchange for a higher overall *AR* (Fig. 2*E*). The relaxation of design constraints also allowed for the design of double-FMN aptamer switches, a “cheat” strategy that allows designs to exhibit *AR* values above the thermodynamic limit for a single FMN-bound switch (*SI Appendix*, Fig. S3).

Computational predictions of RNA secondary structures for top-performing switches revealed the particular utility of interleaving base pairs between the FMN and MS2 aptamers in the inactive state (misfolded reporter) and of “miniaturizing” designs by promoting stable base pairings between the 5′ and 3′ regions or nonswitching stems and hairpins that sequester nonswitching nucleotides (Fig. 2 *B* and *F*). To validate these structural mechanisms, we carried out SHAPE chemical mapping (25) and single-mutant as well as compensatory rescue (26) analysis for a top-scoring design “JL-sng2-3.09.” These data confirmed the predicted secondary structures in both ligand-free and ligand-bound states (Fig. 2*G* and *SI Appendix*, Fig. S4). To further test the general usability of these sensors in different experimental contexts, we shifted our readout from RNA-MaP to cytometric analysis of RNA switches displayed on micromagnetic particles (27). Using this orthogonal assay, JL-sng2-3.09 exhibits robust switching behavior across a wide dynamic range of both FMN and MS2 concentrations, suggesting that these designs are transferrable to other assays (Fig. 2*H*).

The high performance of player-designed switches encouraged us to compare a concurrently developed, physics-based computational algorithm for RNA switch design—Ribologic (19)—against the performance of the Eterna community. In the FMN sensor challenges, Ribologic designs exhibited lower *AR*s than the final player designs after iterative refinement (Fig. 2*A*). To compare Ribologic designs with player-curated designs prior to iterative refinement, we performed an additional round of MS2-activated small molecule switches using RNA-MaP technology. Here, we introduced previously unused small molecule aptamers that bind theophylline and l-tryptophan and challenged players and Ribologic to design diverse on and off switches (small molecule binding generates a fluorescence signal for “on” sensors and decreases the fluorescence signal for “off” switches).

**Fig. 3.**
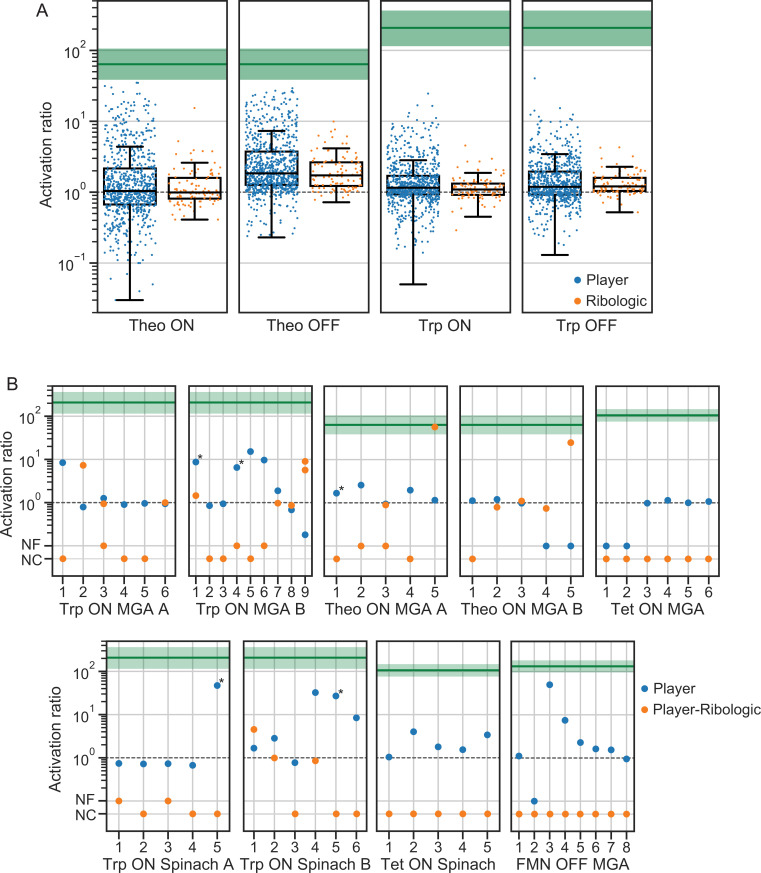
Single-round design challenges on alternative inputs and output. (*A*) Box plots (IQR with whiskers at ±1.5 IQR) show that player designs of both on and off switches coupling the theophylline (Theo) and tryptophan (Trp) aptamers to MS2 coat protein binding exhibited significantly higher *AR*s than Ribologic-designed solutions. Theo solutions approached the thermodynamic limit for *AR*s (depicted as green lines; estimated from measured intrinsic ligand aptamer *K*_d_ values). (*B*) Light-up round designs for Trp, Theo, tetracycline (Tet), and FMN input ligands coupled to folding of malachite green (MGA) or Spinach light-up aptamers. Each vertical line represents a different switch architecture, designed by a player, that was used as a starting point for the corresponding Ribologic design in the same column. Some player designs were used to seed more than one Ribologic solution. NF indicates that the tested design was nonfunctional in both states (no detectable binding to fluorophore); NC implies that Ribologic was unable to converge to a solution for the given design. *Designs confirmed across multiple independent measurements, with the mean *AR* displayed.

In each of these eight puzzles, the best crowdsourced designs outperformed Ribologic-generated designs by consistently achieving *AR* values above 10 and in several cases approaching the thermodynamically allowed maximum (Fig. 3*A* and *SI Appendix*, Fig. S2). We note that Eterna performance was benchmarked against a single competing algorithm, which is a limitation of this study. We also note that in general, there is decoupling between the performance of RNA switches based on our in silico energy calculations and the measured performance of these switches (19) in our assay, suggesting that algorithms that use thermodynamic RNA folding parameters to model switch capability may have fundamental challenges identifying high-performing switches.

Next, we asked if players could apply the lessons learned from past rounds to produce winning solutions when given dramatically fewer overall experimental attempts. To prevent modifications of past successful designs, we introduced two previously unused reporting aptamers—the malachite green aptamer (MGA) (28, 29) and the 3,5-difluoro-4-hydroxybenzylidene imidazolinone (DFHBI)-binding aptamer RNA Spinach (30)—that bind to and activate the fluorescence of otherwise nonfluorescent small molecule dyes and designed puzzles that coupled small molecule aptamers against tryptophan, theophylline, and FMN as well as tetracycline (31). For these “light-up” challenges, we reduced the number of tested designs to fewer than 10 per puzzle, solicited solutions through biweekly community-wide voting, and used solution-phase fluorescence as a readout. Despite having dramatically fewer attempts (only 72 designs were tested over all light-up challenges), players continued to deliver highly responsive switches (Fig. 3*B*), with several *AR* values exceeding 15. Attempts to use Ribologic to generate computational solutions to these challenges failed.

We next asked if player guidance could improve the automated design of RNA switches. We used player solutions to seed the designs for player–Ribologic hybrid designs. To do this, we fixed the location of the included aptamer motifs and allowed Ribologic to converge on new designs seeded with player-submitted solutions. In two examples, coupling player-guided aptamer placement with the Ribologic design infrastructure yielded switches surpassing the best player designs, providing an existence proof of a system wherein experience-guided aptamer and reporter placement can enable semiautomated design of highly functional molecular sensors and suggesting routes to approach next-generation RNA switch design algorithms (Fig. 3*B*).

**Fig. 4.**
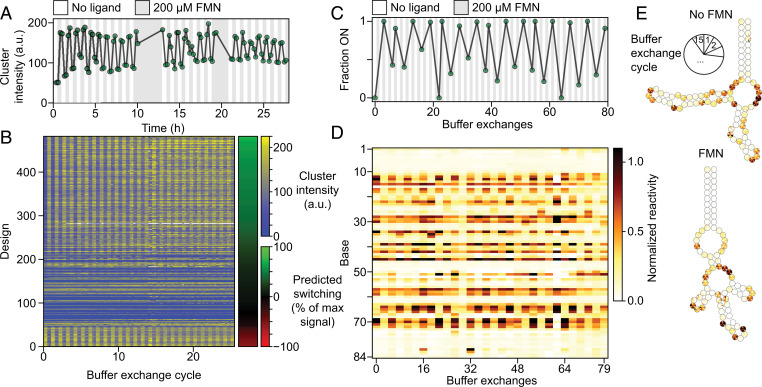
Reversible switching of RNA switches (*A*) Fluorescence signal in arbitrary units (a.u.) from the top-scoring single-aptamer design as the FMN concentration is repeatedly toggled from 0 (white) to 200 µM (gray). Signals from up to 112 individual clusters were averaged. (*B*) FMN switches from the same experiment in *A* ordered by predicted switching magnitude and direction (right bar). Bottom constructs are predicted to switch in the opposite direction (red color in the right bar). (*C*) Switch behavior across 80 buffer exchanges as probed by chemical mapping (dimethyl sulfate probing carried out on aliquots after approximately every third exchange, as indicated by the green points in the graph). (*D*) Reactivity per base for the aptamer profiled in *C* (each point in *C* corresponds to a column in *D*). (*E*) Reactivity per base mapped onto predicted secondary structures. Each base is divided into 15 segments corresponding to each time point.

An important and previously undemonstrated goal for RNA design is the reusability of designed RNA switches to enable energy-neutral applications. In our final experiments, we therefore tested the reversibility of switches by exposing 482 FMN–MS2 Eterna designs to 52 buffer exchanges (cycling between 0 and 200 μM FMN) over 29 h. In all switches, we observed consistent toggling of the fluorescence output (Fig. 4 *A* and *B*), with many designs exhibiting only minor functional degradation over the length of the assay—suggesting near-full reversibility. To test switching in the absence of the MS2 reporter molecule, we immobilized the JL-sng2-3.09 design on DNA-coated beads and carried out 80 FMN buffer exchanges, subjecting aliquots at each point to dimethyl sulfate chemical mapping to assess RNA conformational changes. We observed toggling of the global RNA structure through the entire time course (Fig. 4 *C–E*). Finally, we measured the kinetics of activation for two tryptophan-responsive light-up sensors coupled to the MGA and Spinach aptamers. Both yielded design-specific responses to their respective ligands on the order of seconds to minutes, confirming their suitability for kinetic measurements on the timescale of tens of minutes or better (*SI Appendix*, Fig. S5).

To understand how top Eterna players achieved success in designing efficient stand-alone riboswitches, the community engaged in “all-hands” online discussions, presentations during annual Eterna conferences, and compilation of puzzle-solving strategies in game forum posts and widely shared Google documents. As expected, players approached new puzzles in a highly varied manner, but several recurring principles were articulated.

First, no single puzzle architecture was universally suitable for all given input–output combinations. Rather, the testing of various puzzle architectures that differed in the relative placement of the input and output aptamers was required for identifying high-efficiency switches. For the first FMN–MS2 on-switch puzzles (Fig. 2), many successful solutions placed the FMN input aptamer and the MS2 output hairpin as close as possible to one another and sequestered other nucleotides available for design into “static stems.” Several independent solutions converged on an architecture with identical ordering of the FMN aptamer segments, MS2 segments, and static stems up to circular permutation of the sequence (Fig. 2*G*). Light-up sensor challenges involved larger aptamer sequences than the FMN aptamer, and both the input ligand aptamer and the output fluorophore aptamer involved two segments that could be placed by designers. New rules were proposed for these more difficult challenges. While the most successful player designs all included a “split” aptamer (where one aptamer is nested inside the other), determining which aptamer was split to yield optimal performance was often determined by considering the affinity of the aptamer (the higher-affinity aptamer was generally split), the individual aptamer sequences (aptamers predicted to fold stably were often split), and any sequence similarity that may exist between the two aptamers. Exceptions to these general rules are also apparent, highlighting that optimal switch design requires testing not only multiple switch designs but also multiple switch architectures.

Second, in initial FMN–MS2 challenges that allowed for iterative refinement of solutions, players commonly modified the most successful designs in every round in order to improve performance in subsequent rounds, leading to design “families” sharing high sequence similarity (Fig. 2 *B* and *C*). In addition, the most successful designs were confirmed to switch in silico using multiple folding energy calculations. While the Vienna RNA 1.8.5 folding package was originally the de facto RNA folding engine for Eterna, veteran Eterna players suggested that engines such as Vienna 2.0 and NuPACK were more predictive and that the best switches were predicted to function in all three engines. For this reason, we provided players in-game tools to model their switches in multiple folding engines in all later rounds of puzzles.

Finally, several players spearheaded the development and dissemination of tools that granted insights into individual switch puzzles and/or solutions. During light-up challenges, players developed visualization tools for simultaneously tracking both states of the sensor and tabulating metrics that were not provided in the Eterna interface. For example, one metric was a solution’s “x ratio.” This is the ratio of predicted pairing probabilities in the absence vs. presence of the input ligand of the outermost base pairs of the split aptamer, which approximately predicts the actual *AR* of the design. Players generally sought a high predicted x ratio, and this feature was incorporated into a player-designed “arcplot” online tool that showed pairing probabilities of all bases in the absence and presence of the ligand in a single chart. Players also disseminated screenshots of arcplots with striking features (e.g., spirals) (*SI Appendix*).

This study demonstrates that an iterative, massive open laboratory approach—internet-scale crowdsourcing of experimental hypotheses in combination with a high-throughput, quantitative experimental pipeline for hypothesis testing—enables design of near–optimally efficient, reversible, self-contained RNA switches that couple diverse input molecules to diverse output fluorescence modalities, opening the door to a variety of applications. We believe that other citizen science projects would benefit by enabling their communities to define and test all incoming hypotheses (6). More specific to the field of synthetic RNA biology, we found it critical to explore diverse motif orderings and structure-toggling mechanisms to achieve optimal performance. The merits of such exploration have not been broadly appreciated by the RNA design field and are likely applicable to other unsolved problems in synthetic biology. Furthermore, we are optimistic that the ongoing revolution in high-throughput biology will engender the creation of other platforms “democratizing” experimental biological science through the provisioning of low-cost experimental validation of hypotheses generated by diverse nonexperts.

## Materials and Methods

RNA molecules were designed in Eterna (https://eternagame.org). For RNA-MaP high-throughput functional assays, Eterna participants’ solution sequences were collected in a series of rounds, and oligonucleotides were synthesized corresponding to these solutions, bottlenecked, and then sequenced. Sequencing chips were recovered and mounted on a custom automated fluorescent microscope, and they were quantified for reporter binding across multiple ligand concentrations. Fluorescence images were aligned to the sequencing data; individual cluster intensities were quantified with a custom analysis pipeline, normalized, and fit to a binding curve, and *AR* values were calculated. Detailed materials and methods, including descriptions of *AR* quantification and ancillary quantification methods, are in *SI Appendix*, *Materials and Methods*. Data are also archived on the Eterna website (https://eternagame.org). Control and analysis software is available at https://github.com/GreenleafLab.

## Supplementary Material

Supplementary File

Supplementary File

## Data Availability

The Eterna results are available on the Eterna website (https://eternagame.org) and included as Dataset S1. Control and analysis software has been deposited in GitHub (https://github.com/GreenleafLab).

## Change History

May 4, 2022: The article license has been updated.
