# Supplementary material for: Crowdsourced RNA design discovers diverse, reversible, efficient, self-contained molecular switches
Source: Proc Natl Acad Sci U S A. 2022 Apr 26;119(18):e2112979119. doi: 10.1073/pnas.2112979119 (PMC9170038; doi:10.1073/pnas.2112979119)
Supplement: Supplementary File [file pnas.2112979119.sapp.pdf]

Supplementary Materials for

Crowdsourced RNA design discovers diverse, reversible, efficient, self-contained molecular switches

Johan O. L. Andreasson[1,2], Michael R. Gotrik[2], Michelle J. Wu[3] , Hannah K. Wayment-Steele[4], Wipapat Kladwang[2], Fernando Portela[2,5], Roger Wellington-Oguri[2,5], Eterna Participants[5], Rhiju Das[2,6,*], William J. Greenleaf[1,7,8,*]

[1]Department of Genetics, Stanford University School of Medicine, Stanford University, Stanford, California 94305, USA.
[2]Department of Biochemistry, Stanford University School of Medicine, Stanford University, Stanford, California 94305, USA.
[3]Biomedical Informatics Training Program, Stanford University School of Medicine, Stanford University, Stanford, California 94305, USA.
[4]Department of Chemistry, Stanford University, Stanford, California 94305, USA.
[5]Eterna Massive Open Laboratory.
[6]Department of Physics, Stanford University, Stanford, California 94305, USA
[7]Department of Applied Physics, Stanford University, Stanford, California 94305, USA
[8]Chan-Zuckerberg Biohub, San Francisco, CA, USA

*Correspondence to: R. Das (e-mail: rhiju@stanford.edu) & W. Greenleaf (e-mail: wjg@stanford.edu).

Materials and Methods

Eterna Online interface

RNA molecules were designed in Eterna (https://eternagame.org), an online platform where participants solve design challenges, or puzzles, directly in a web browser(*1*). The game-like interface was updated with improved design and features that help participants, such as indicators for design constraints and a 'stamper' tool that allows for quick interactive entry of predefined sequences, such as the MS2 RNA hairpin. The range of available RNA folding engines was expanded from ViennaRNA 1.8.4(*2*) to also include ViennaRNA package 2.1.9(*3*) and NuPACK 3.0.4(*4*), and participants could toggle between these engines during design. Designs were not required to fold or switch correctly in any of these computational folding engines to be submitted by participants.

High-throughput analysis of participant-designed switches by RNA-MaP

Sequencing libraries

For RNA-MaP high-throughput functional assays, Eterna participants' solution sequences were collected in a series of rounds. Within the global round numberings across all Eterna challenges, sequences for this study were collected in R88 to R107; see Fig. S1 and Tables S1-S2. These sequences were prepended and appended with common flanking sequences (see below) and synthesized (CustomArray, Bothell, WA), using the procedure recently described(*5*). Briefly, sequencing libraries were prepared from the oligo pool either by PCR or emulsion PCR (ePCR)(*6*, *7*) in multiple steps, as follows. First, the oligo pool was amplified with primers Read2 and RNAPstall or RNAPstall2. In the second step, sequences for Illumina sequencing adapters (C_adapter and D_adapter), sequencing primers (Read1 and Read2), RNAP promoter and stall sequences, and, for later Eterna rounds, a 16-nt barcode sequence, were added by PCR. Concentrations were 1.5 nM for the previously amplified oligo pool, 3.8 nM for the flanking DNA oligos (C_Read1_RNAPstall and D_Read2) and 137 nM for outer primers (C_adapter and D_adapter). The optimal number of cycles was determined by gel electrophoresis. For libraries prepared by ePCR, the oligo pool was amplified in one step, as previously described(*7*).

For libraries with barcodes, a bottlenecking step was performed. The library was diluted to ~700,000 molecules and amplified for 16 cycles with primers C_adapter and D_adapter . Libraries were quantified by qPCR or Qubit (Thermo Fisher Scientific) and sequenced on an Illumina MiSeq sequencer with 150-cycle kits.

Oligonucleotide sequences for RNA display

Primer name	Sequence
Read2	CGGCATTCCTGCTGAACCGCTCTTCCGATCT
RNAPstall	GTAAGGAGGTTGTATGGAAGACGTTCCTGGATCC
RNAPstall2	GTAAGGAGGTTGTATGGAAGACGTTCCTGGAT
C_adapter	AATGATACGGCGACCACCGAGATCTACAC
D_adapter	ATCTCGTATGCCGTCTTCTGCTTG
C_Read1_RNAPstall	AATGATACGGCGACCACCGAGATCTACACTCTTTCCCTACACGACGCTCTTCCGATCTTTTATGCTATAATTATTTCATGTAGTAAGGAGGTTGTATGGAAGACGTTCCTGGATCC
C_Read1_RNAPstall2	AATGATACGGCGACCACCGAGATCTACACTCTTTCCCTACACGACGCTCTTCCGATCTTTTATGCTATAATTATTTCATGTAGTAAGGAGGTTGTATGGAAGACGTTCCTGGAT
C_Read1_BC_RNAPstall	AATGATACGGCGACCACCGAGATCTACACTCTTTCCCTACACGACGCTCTTCCGATCTNNNNNNNNNNNNNNNNTTTATGCTATAATTATTTCATGTAGTAAGGAGGTTGTATGGAAGACGTTCCTGGAT
D_Read2	CAAGCAGAAGACGGCATACGAGATCGGTCTCGGCATTCCTGCTGAACCGCTCTTCCGATCT

Preparation and measurement of standalone RNA switches on an Illumina sequencing chip

Sequencing chips were recovered and mounted on a custom automated fluorescent microscope, as previously described(*8*). The binding of a SNAP-549-labeled MS2 coat protein (MS2-dlFG mutant) to each RNA design, in the absence or presence of its target small molecule was measured directly on the flow cell, similar to our previous studies(*9*). Briefly, the sequencing chip was recovered and mounted onto a custom sequencer-based microscope where dsDNA templates were generated from the clonal sequencing clusters. Clusters of clonal ssDNA strands were recovered by stripping residual fluorophores and complementary DNA after sequencing. A biotinylated oligonucleotide was hybridized to the ssDNA, and dsDNA templates were generated by Klenow extension. Common sequences were further blocked and streptavidin was added to generate a roadblock at the end of the template. *E. coli* RNAP was initiated and stalled by nucleotide starvation, excess polymerase was removed, and the full design was then generated at saturating nucleotide concentrations for 10 min. The common RNA sequences representing the RNAP stall sequence and the Read2 primer were blocked by hybridizing Cy5-labeled and unlabeled DNA oligonucleotides, respectively, at concentrations of 500 nM during transcription and then in buffer for an additional 10 min. MS2 protein was introduced to the flow cell, starting at 0.7 or 1.5 nM, and increased by factors of two up to 3 µM. Incubation times varied from 0.8–1.5 h at the lowest concentrations to 10–20 min at the highest concentrations. The buffer conditions for RNA-MaP titrations were 100 mM Tris-HCl, pH 7.5, 80 mM KCl, 4 mM $MgCl_2$, 0.1 mg/mL BSA, 1 mM DTT, 0.01 mg/mL yeast tRNA, 0.01% Tween-20. Subsequently, the DNA was stripped, RNA regenerated for each cluster, and the MS2 titration repeated in the presence of constant amounts of each small molecule ligand. Fluorescence images of the generated RNA and the bound MS2 were collected using red (660 nm, 200 mW, 400 ms exposure time) and green lasers (532, 200 mW, 400 ms or 1.0 s), respectively. Each design was represented by multiple clusters (average N_{cluster} = 32–84, for early rounds; see Figure S1) with reproducibility improving with the use of random barcode sequences and a bottlenecking step (N_{cluster} = 57–191, for rounds see Figure S1).

Data Fitting

Binding curves for MS2 protein binding (at protein concentrations 1–3000 nM) were collected for all clusters (e.g. **Fig. 1e**) in the presence and absence of the relevant small molecule ligand (e.g., 0 and 200 µM FMN). Fluorescence images were aligned to the sequencing data and individual cluster intensities were quantified with a custom analysis pipeline, normalized, and fit to a binding curve. Experimental reproducibility was determined by including a control set of one thousand standard designs in each Round, which exhibited excellent agreement between replicates (Fig S1).

The magnitude of switching of the designs was determined by the fold-change in the effective dissociation constant, K_D, for MS2 protein reporter binding in response to small-molecule ligand binding. The binding should be strong (low K_D) in the ON-state, where the RNA should display the reporter-binding motif, and weak (high K_D) in the OFF-state without the motif formed. In contrast to an activation ratio measured at a fixed reporter concentration, the fold change in K_D is derived from all available data, measures changes from the full range of ON-state reporter affinities, and can be directly related to biophysical models. Furthermore, the fold change in K_D gives the change in activation at lowest reporter (output) ligand concentrations, where it should be maximal(*10*).

Data analysis

For each image, the cluster positions were aligned to the sequencing data coordinates and quantified using a custom analysis pipeline, as previously described(*5*). The green (MS2) signal was normalized to the red (RNA) signal for each RNA cluster. The fluorescence signals for MS2-only controls were fit to a binding curve

$$F([MS2]) = F_{max}\frac{[MS2]}{[MS2]+K_D} \qquad (1)$$

Where $F_{\max}$ is the maximum fluorescence intensity, [MS2] is the MS2 concentration and K_D is the observed dissociation constant. All control clusters were fit individually and the median $F_{\max}$ was then used to further normalize all cluster signals. These signals were then fit to the same binding curve as above and bad clusters were filtered out and the median K_D and $F_{\max}$ values for each design were used for further analysis and for reporting data back to Eterna participants.

Scoring of participant designs

Full experimental data reports were returned to the Eterna community for each tested design. In addition, to aid in tracking progress, each experimentally tested Eterna design was given a single summary "Eterna Score" (on a scale of 0–100), based on sum of three subscores, as follows.

The Switch Subscore (*SS*) was calculated as

$$SS = 40\,Max\left(0, Min\left(1, log\,(FC)\,/log\,(FC_{high}\right)\right)$$

Here *FC* is the activation ratio (originally termed 'fold-change'), the ratio in the observed MS2 dissociation constant, $K_{D,\,obs}$, for the design and FC_{high} is an upper bound that represents the maximum signal. For the MS2 switches in this study, we originally set $FC_{high} = 26$. This value corresponded to the maximum activation ratio achievable based on estimates of the dissociation constant of the FMN aptamer (which were overestimated), FMN ligand concentration, and a desired $K_{D,\,obs}$ in the ON state of within 2-fold of the dissociation constant for the MS2 protein to a perfect aptamer ($K_{D,obs,ON} < 2\ K_{D,MS2}$). (To avoid confusion in scoring, we maintained the value of $FC_{high} = 26$ even as we improved measurements of the FMN aptamer dissociation constant; instead we noted to players in puzzle descriptions that activation ratios as high as 100 should be achievable and sought.)

The Baseline Subscore (*BS*) was calculated as

$$BS = 30\,Max\left(0, Min\left(1, 1.5 - 0.25\frac{K_{D,obs,ON}}{K_{D,MS2}}\right)\right)$$

where $K_{D,obs,ON}$ is the observed dissociation constant in the ON-state and $K_{D,MS2}$ is the affinity for the native MS2 hairpin, i.e., the score is maximized for $K_{D,obs,ON} < 2\ K_{D,MS2}$ and then decreased linearly with $K_{D,obs,ON}$ until being 0 for $K_{D,obs,ON} > 6\ K_{D,\,MS2}$. This subscore penalized switches whose ON configurations bound MS2 coat protein significantly worse than an isolated MS2 RNA hairpin. The value of $K_{D,\,MS2}$ was measured through control MS2 hairpin RNA sequences included in every experiment and fell in the range of 1-2 nM.

The Folding Subscore (*FS*) was

$$FS = 30\, Max\left(0, Min\left(1, \frac{(F_{max} - 0.3)}{0.4}\right)\right)$$

such that it was 0 for $F_{max} < 0.3$, 30 for $F_{max} \geq 0.7$, and linearly increasing in between. This subscore penalized RNA sequences that misfolded in their ON states and prevented MS2 protein binding.

Optimal activation ratio set by equilibrium thermodynamics

Throughout our study, we compared experimentally achieved activation ratios (AR; originally termed fold-changes, FC) with thermodynamically optimal ARs. The optimal AR of a standalone RNA switch depends upon the affinity of the aptamer for the input ligand and the concentration of input ligand used to trigger the switch. A detailed description of this bound is given in reference[7], but for completeness, a brief derivation is given here.

As an example, we consider an FMN-controlled OFF-switch standalone RNA switch construct controlling the formation of an MS2-binding hairpin aptamer, the expression platform used for the standalone RNA switches in this work. The thermodynamic efficiency of this construct can be estimated as follows. The partition function of the system is given by partitioning the system's full conformational ensemble into four states with the following conformations and energies (*E*):

State	FMN aptamer formed	FMN bound	MS2 hairpin formed	MS2 bound	E
**1**	y	y	n	n	$-k_B T \ln\left(\frac{[FMN]}{K_{D,FMN}}\right)$
**2**	y	n	n	n	0
**3**	n	n	y	n	ΔG
**4**	n	n	y	y	$\Delta G - k_B T\, ln\left(\frac{[MS2]}{K_{D,MS2}}\right)$

Here, k_B is Boltzmann's constant, T is the absolute temperature, [*FMN*] and [*MS2*] are the experimental concentrations of FMN and MS2, respectively, and ΔG is an unknown energy difference between the states without bound FMN or MS2. The fluorescent signal is only observed in state **4** and the probability of being in this state, derived from the partition function, is

$$p(signal) = \frac{[MS2]}{([MS2] + K_{D,obs})},$$

where

$$K_{D,obs} = K_{D,MS2}\left(1 + e^{\frac{\Delta G}{k_B T}}\left(1 + \frac{[FMN]}{K_{D,FMN}}\right)\right)$$

Accordingly, the normalized signal looks identical to a canonical binding curve. For ON-switches, a similar five-state model yields an analogous functional form, with

$$K_{D,obs} = K_{D,MS2} \frac{\left(1 + e^{\frac{\Delta G}{k_B T}}\right)\left(1 + \frac{[FMN]}{K_{D,FMN}}\right)}{\left(1 + e^{\frac{\Delta G}{k_B T}} + \frac{[FMN]}{K_{D,FMN}}\right)}.$$

For the constructs evaluated in this work, the reported ARs represent the ratio between the observed dissociation constants, $K_{D,obs}$, at two experimental ligand concentrations (for instance, 0 and 200 µM for FMN). In either ON or OFF switches, this ratio limits to $1 + \frac{[FMN]}{K_{D,FMN}}$ as the Boltzmann-weighted term involving ΔG becomes large. We may see this in the following expression for the AR of an OFF-switch:

$$\frac{K_{D,obs}([FMN]=200)}{K_{D,obs}([FMN]=0)} = \frac{1+e^{\frac{\Delta G}{k_B T}}\left(1+\frac{[FMN]}{K_{D,FMN}}\right)}{1+e^{\frac{\Delta G}{k_B T}}} < 1 + \frac{[FMN]}{K_{D,FMN}} = AR_{max}.$$

The expression for an ON-switch follows similarly. We refer the reader to ref.[7] for a more complete analysis of this thermodynamic model.

Independent assessment of an MS2 standalone RNA switch using flow cytometry

As an independent test of an FMN-MS2 standalone RNA switch discovered through RNA-MaP measurements, we displayed the RNA on particles to enable characterization of bound MS2 through flow cytometry. The sequence used was:

BC_ JL-sng2-3.09 (underline is the active region)

GGGUAUGUCGCAGAA<u>GUAGCUAUCGGAGGAUAUUCAUACCGGAAACGGACAUGAGGAUCACCCA</u>
<u>UGUGGCGAAAGCCUUGGGAGAAGGCUGAUAGCUAC</u>AACUGCGACAUACCCAAAAAAAAAAAAAA
AAAAAAAAAAA

Primers used for BC_ JL-sng2-3.09 assembly

BC_ JL-sng2-3.09_P1	CGACAGCAGTTCTAATACGACTCACTATAGGGTATGTCGCAGAAGTAGCTATC
BC_ JL-sng2-3.09_P2	GTGATCCTCATGTCCGTTTCCGGTATGAATATCCTCCGATAGCTACTTCTGCGACATACC
BC_ JL-sng2-3.09_P3	GGAAACGGACATGAGGATCACCCATGTGGCGAAAGCCTTGGGAGAAGGCTGATAGCTACA
BC_ JL-sng2-3.09_P4	TTTTTTTTTTTTTTTTTTTTTTTTTGGGTATGTCGCAGTTGTAGCTATCAGCCTTCTCCC

BC_MG-D3 (control sequence containing same flanking sequence)

GGGUAUGUCGCAGAA<u>GGGACACAAUGGACGCAGAUAAUCGGAUUCCCGACUGGCGAGAGCCAGG</u>
<u>UAACGAAUGGGCCGGUUUCUGUGUUAACGGCCG</u>AACUGCGACAUACCCAAAAAAAAAAAAAAAA
AAAAAAAAA

Primers used for BC_MG-D3 assembly

BC_JL_P1	CGACAGCAGTTCTAATACGACTCACTATAGGGTATGTCGCAGAAGGGACACAATGGACGC
BC_JL_P2	CATTCGTTACCTGGCTCTCGCCAGTCGGGAATCCGATTATCTGCGTCCATTGTGTCCCTT

BC_JL_P3	GAGAGCCAGGTAACGAATGGGCCGGTTTCTGTGTTAACGGCCGAACTGCGACATACCCAA
BC_JL_P4	TTTTTTTTTTTTTTTTTTTTTTTTTGGGTATGTCGCAGTTCGGCCGTTAACA

DNA primer oligos were ordered from Integrated DNA Technologies (IDT) and full-length DNA oligos were assembled using standard PCR assembly protocols available at primerize.stanford.edu(*11*). Briefly, 100 µL of 1X PCR mix containing Phusion DNA polymerase (Thermo Fisher Scientific) was prepared with 2 µM of primers P1 and P4, and 40 nM of primers P2 and P3. Then, 20 cycles of PCR were run at 98 °C (15 s) – 57 °C (30 s) – 72 °C (30 s). The correct length products were verified on agarose gels and transcribed using a Transcriptaid T7 High Yield transcription kit (Thermo Fisher Scientific) using the manufacturer's protocol. After DNase treatment for 30 minutes at 37˚C, RNA was purified using the RNA Clean & Concentrator kit (Zymo Research), resuspended in DEPC-treated H_2O and the RNA concentration was determined using a UV spectrophotometer (NanoDrop, Thermo Fisher Scientific).

We prepared RNA on beads for flow cytometry as follows. The following oligo was ordered from the Protein and Nucleic Acid (PAN) Facility at Stanford University: /5`C6AmM/TTTTT TTTTT TTTTT TTTTT TTTT /3`InvdT/. This poly-T oligo contains an amino modification with a 6-carbon spacer on its 5' end, and was terminated using a 3' inverted deoxythymidine. 100 µL of 2.8 µm carboxylic acid beads (Dynabeads M-270, Invitrogen) were washed once with 100 µL 0.01 M NaOH and twice with 100 µL H_2O. The beads were incubated overnight with rotation at room temperature in 1X conjugation buffer (200 mM NaCl, 1 mM imidazole), 0.01% sodium dodecyl sulfate, 250 mM 1-ethyl-3-(3-dimethylaminopropyl) carbodiimide (EDC) and 100 µM of amino-modified poly-T oligo in 50% v/v DMSO. After incubation, the beads were washed twice with TT buffer (50 mM Tris-HCl pH 7.5, 0.5% Tween-20) and stored in 100 µL TNaTE buffer (10 mM Tris-HCl pH 7.5, 140 mM NaCl, 0.1% Tween-20, 1 mM EDTA) at 4 °C. 7 µL of poly-T-coated beads from above were added to 20 µL PBSMT (1X PBS, pH 7.2 (Gibco), 5 mM $MgCl_2$, 0.01% Tween-20) containing 2 µM of BC_JL RNA or BC_MG-D3 (control) and incubated at 37˚C for 5 minutes and then put on ice for 5 minutes. The buffer was removed and the beads were resuspended in 360 µL PBSMT. 500 uL of 2.4x solutions of FMN (1.944 mM) and SNAP-549-labelled MS2 coat protein (MCP, 1.944 µM) were prepared in PBSMT and these underwent 1:4 dilutions a total of five times. Assays were prepared using 25 µL of each 2.4x solution or PBSMT, as well as 10 µL of RNA-coated beads (to give a total volume per tube of 60 µL) such that the final concentrations ranged from 810 µM to 3.2 µM and 0 µM for FMN and from 810 nM to 3.2 nM and 0 nM for MCP. Each sample was analyzed using a Sony SH800S Cell Sorter and data for 10,000 events were collected per sample. Beads were excited using a 561 nm laser and their emitted fluorescence was measured from the 600±60 nm emission channel.

Fluorescence measurements for light-up sensor challenges

Small molecules for light-up sensor challenges

We developed Eterna puzzles that incorporated previously-described aptamers against the small molecules theophylline (Theo), flavin mononucleotide (FMN), tryptophan (Trp), and tetracycline (Tet), as inputs; and malachite green (MG) and 3,5-difluoro-4-hydroxybenzylidene imidazolinone (DFHBI), as outputs. All molecules were purchased from Sigma-Aldrich unless otherwise specified. The malachite green aptamer (MGA) and DFHBI aptamer (RNA Spinach) belong to a class of light-up RNAs that effect >1000-fold fluorescence enhancement upon

binding the target fluorophore molecule. Specifically, our puzzles coupled small molecule (input) binding to turn-on fluorescence activation for the following input/output pairs: Theo/MG, FMN/MG, Trp/MG, Trp/DFHBI, Tet/MG, and Tet/DFHBI. Pilot experiments with FMN/DFHBI puzzles showed significant fluorescent overlap between FMN and DFHBI that precluded characterization, and these were not continued. In addition, experiments with Theo/DFHBI and ATP/DFHBI sensors showed interference of the aromatic input ligands with RNA Spinach, likely through competitive binding with DFHBI, and these were also not pursued.

In each light-up sensor round, we collected 5-6 participant designs from two puzzles at a time. The characterized designs were chosen by participants in a weekly community-wide vote, appended with complementary flanking sequences, ordered as DNA oligos (Integrated DNA Technologies) designed with Primerize(*11*), PCR amplified using standard protocols, transcribed using a TranscriptAid T7 High Yield Transcription Kit (ThermoFisher), and purified using an RNA Clean & Concentrator-25 kit (Zymo Research) using the manufacturer protocols.

Light-up fluorescence assays

Assays were prepared in 96-well, half-area, flat-bottomed plates (Costar). Each participant design was titrated at 8 different concentrations of their respective light-up dye both in the absence and presence of their target small molecule (see table below for input ligand concentrations). The light-up dyes were titrated from 10 μM to 16 nM in an 8-point, 1:2.5 dilution series, except for in the first light-up series where the dye was titrated from 5 μM to 40 nM in an 8-point, 1:2 dilution series. The final RNA concentration in each well was 200 nM at a final volume of 100 μL. Concentrations for small molecules were obtained from their extinction coefficients:

Molecule	λ_{max}	$\epsilon\ (cm^{-1}M^{-1})$	Concentration
**Theophylline**	277	10,200	1.2 mM
**Tryptophan**	278	5,579	2.4 mM
**Tetracycline**	366	14,150	60 μM
**FMN**	450	9,910	200 μM
**DFHBI**	405	11,864	varied
**MG**	617	148,900	varied

After equilibration of the wells for 30 minutes at room temperature, fluorescence intensity for each well was measured using a Tecan Infinite 200 PRO plate reader. For switches containing the Spinach aptamer to DFHBI, excitation was at 466 $\pm$ 9 nm and emission measured at 508 $\pm$ 20 nm. For switches containing the MGA aptamer to malachite green, excitation was at 625 $\pm$ 9 nm and emission measured at 660 $\pm$ 20 nm.

Data Fitting

The resulting fluorescence intensities in each well were plotted against the light-up (LU) dye concentration and fit to the equation

$$B = B_{max} * \frac{[LU]}{[LU] + K_D^{LU}}$$

Where B is the brightness at each concentration [LU] of the respective light-up dye, B_{max} is the brightness at saturation for the given sample, and K_D^{LU} is the dissociation constant for the design to the dye. To determine experimental activation ratios, we determined the ratio of LU fluorescence without and with ligand at lowest LU ligands, analogous to the RNA-MaP measurements above. More specifically, for each design, we determined the B_{max} and K_D in both the absence (-) and presence (+) of the input ligand. We determined the activation ratio of each design by comparing the signal in the presence and absence of input ligand:

$$AR = \frac{B^{(+)}}{B^{(-)}} = \frac{B_{max}^{(+)}}{B_{max}^{(-)}} * \frac{[LU] + K_D^{LU(-)}}{[LU] + K_D^{LU(+)}}$$

Which, in the case for an ON switch where $K_D^{LU(-)} > K_D^{LU(+)}$ approaches its maximum at low [LU]:

$$AR_{.}^{LU\to 0} = \frac{B_{max}^{+SM}}{B_{max}^{-SM}} * \frac{K_D^{LU(-)}}{K_D^{LU(+)}}$$

The activation ratio of each design was then compared to its theoretical max

$$AR_{max} = \frac{[input]}{K_d} + 1$$

Where the K_d value of the switch to the respective small molecule was determined using DMS Chemical Mapping under identical buffer conditions and using a non-switching ("always ON") ligand-binding control sequence of similar architecture to the respective puzzle.

Data Analysis

The K_D^{LU} and B_{max} values were computed for each switch design in the presence or absence of the respective small molecule. Unlike the MS2 switches characterized on the array, the B_{max} value for a given RNA can change based on the predominant state of the switch. That is, two states of the same switch can promote different levels of fluorescence enhancement of the LU dye independently of their affinity to the dye. For this reason, we report three ratios (K_D^{LU}, B_{max}, and $AR_{.}^{LU\to 0}$) for each participant design based on their fit in the absence or presence of the relevant small molecule ligand. Switches whose K_D^{LU} were higher than the limits of detection or whose B_{max} values did not significantly exceed background (i.e., designs where both fitted B_{max} values were >20-fold lower than the K_D^{LU} of the non-switching control light-up aptamer used in each round) were marked as non-functional.

Kinetic characterization of light-up sensors

We wished to determine the rate-limiting step for switching of sequence BH_E029, a Trp/MG "ON" switch through kinetic measurements. We characterized the sequence

```
BH_E029:
GGGAGAGUGGCAGCUUACAACGUAACGAUGCGGCCGCCACUGAUCCGACUGGUUAACAGAAAGUCCUGACCAGGUAA
CGAAUGGUCAGGACCGGGCAUUGUUACGAAGUGGGCUGUUGCUUUUCC
```

Stock 3x solutions of MG (172 nM), RNA (150 nM), and Tryptophan (7.2 mM) were prepared in 1X PBS with 5 mM $MgCl_2$ at pH 6.1 (PBSM). To prepare the pre-equilibrated controls, 30 minutes prior to injection we mixed 30 µL each of 3x MG, 3x RNA, and either 3x Trp (yellow curves) or 1x PBSM (red curves) buffer. To prepare the non-equilibrated controls, we prepared the same mixtures except withholding either RNA (**Fig. S4A**), or Trp (**Fig. S4B**). At the time of measurement, all wells had final reagent concentrations of 64 nM MG, 50 nM RNA, and 2.4 mM Trp in a total volume of 90 µL and all wells were prepared in duplicate.
To determine the rate at which Trp bound to the switch (Figure A), we injected 30 µL of 3x RNA into both of the Trp(+) and Trp(−) wells to yield the final concentration given above and measured the fluorescence intensity of these 4 samples and their pre-equilibrated controls every ~3.3 s for 30 minutes. We then fit the fluorescence (B) as a function of its maximum fluorescence (B_{max}) time (t) and the association (k_{on}) :

$$B = B_{max} * \left(1 - exp\left(-k_{on} \cdot (t_0 + t)\right)\right)$$

Where B represents the fluorescence intensity at each point, B_{max} the maximum brightness at equilibrium, k_{on} the association rate (s^{-1}), t the time from injection (seconds), and t_0 the delay (seconds) between injection and the first measurement (about 6 seconds).

To determine the rate at which MG bound to the switch (Figure B), we injected 30 µL of 3x MG into both of the Trp(+) and Trp(−) wells to yield the same final concentrations as above and measured the fluorescence intensity of these 4 samples and their pre-equilibrated controls every ~3.3 s for 5 minutes. The association rate of MG with the switch was too fast for measurement under these conditions.

Chemical mapping measurements
To test reversible structural toggling of RNA switches by FMN without MS2 coat protein, RNA molecules for the JL-sng2-3.09 sensor were generated by T7 RNA polymerase *in vitro* transcription and purified, as in our previous protocol (*13*). This RNA was initially hybridized to magnetic poly-dT beads (Poly(A) Purist, Ambion) and incubated at 4 °C. Buffer and salt conditions were identical to those in the RNA-MaP experiments (100 mM Tris-HCl, pH 7.5, 80 mM KCl, 4 mM $MgCl_2$) and the FMN concentration was varied by repeatedly pulling down (via magnetic stand) and re-suspending the beads in buffer without or with FMN (0 or 200 µM). Incubation times after each buffer exchange was 5 min, and the experiments were carried out at ambient temperature (24 °C). Every three cycles, two sub-samples (20 µL each) from the large experimental volume (initially 1.28 mL) were collected and chemically probed using 1-methyl-7-nitroisatoic anhydride (1M7) or left unmodified, as controls. Workup of these sub-samples, including purification, reverse transcription, and quantitative analysis in HiTRACE (*12*), followed our previous protocol (*13*).

Measurements of affinities of each RNA aptamer to its target ligand (FMN, tetracycline, tryptophan, theophylline) was also carried out by 1M7 or DMS chemical mapping (see Table S3). The sequences used for these chemical mapping experiments included RNA constructs designed to present the aptamers within stable hairpins, flanking sequences that matched those of

RNA-MaP or light-up fluorescence experiments, as appropriate, and additional 3' sequences used to prime reverse transcription, as follows:

Aptamer	Condition	RNA Sequence
FMN	RNA-MaP	GGAAAGGCGUGU**AGGAUAU**GCUUCGGC**AGAAGG**ACACGCCAAAGAAACAACA<br>ACAACAAC
Tetracycline	RNA-MaP	GGAAAGGCG**CUAAAACAUACC**AGUUCGCU**GGAGAGGUGAAGAAUACGACCAC**<br>**CUAG**CGCCAAAGAAACAACAACAACAAC
Tryptophan	RNA-MaP	GGAAAGGCGAG**CGGCCGCCACU**GUUCGC**AGGACCGG**GCUCGCCAAAGAAACA<br>ACAACAACAAC
Theophylline	RNA-MaP	GGAAAGGCGAG**GAUACCAG**CGUUCGCG**CCCUUGGCAGC**CUCGCCAAAGAAAC<br>AACAACAACAAC
FMN	Light-up	GGGAACGUCUCCGAGUAGGAGACGAAGGGAGAGUGGCAGCUUACAAUUCCAG<br>CCGACCACUGAAAUACUCUAG**AGGAUA**UGACAGACAAAGUCUGUC**AGAAGG**C<br>UAGAGCAGUGGGUAACGAAUGCUGGAAAAGUGGGCUGUUGCUUUUCCAAGCU<br>UAUCGAGUAGAUAAGCAAAAAGAAACAACAACAACAAC
Tetracycline	Light-up	GGGAACGUCUCCGAGUAGGAGACGAAGGGAGAGUGGCAGCUUACAACG**CUAA**<br>**AACAUACC**UGCACCACCGACCUUGAAAACAAGGGUAACGAAUGUGGAGCA**GG**<br>**AGAGGUGAAGAAUACGACCACCUAG**CGAAGUGGGCUGUUGCUUUUCCAAGCU<br>UAUCGAGUAGAUAAGCAAAAAGAAACAACAACAACAAC
Tryptophan	Light-up	GGGAACGUCUCCGAGUAGGAGACGAAGGGAGAGUGGCAGCUUACAAACUACA<br>GUUG**CGGCCGCCACU**GCGAAGGACGGGUCCCUCGAAAGAGGUUGAGUAGAGU<br>GUGAGCGC**AGGACCGGG**CAACAGUAGUAAGUGGGCUGUUGCUUUUCCAAGCU<br>UAUCGAGUAGAUAAGCAAAAAGAAACAACAACAACAAC
Theophylline	Light-up	GGGAACGUCUCCGAGUAGGAGACGAAGGGAGAGUGGCAGCUUACAACCUGAA<br>GGACGGGUCCCCAGUAAUAAU**GAUACCAG**GAACAAAAGUUC**CCCUUGGCAGC**<br>AUCUGGGUUGAGUAGAGUGUGAGCAGGAAGUGGGCUGUUGCUUUUCCAAGCU<br>UAUCGAGUAGAUAAGCAAAAAGAAACAACAACAACAAC

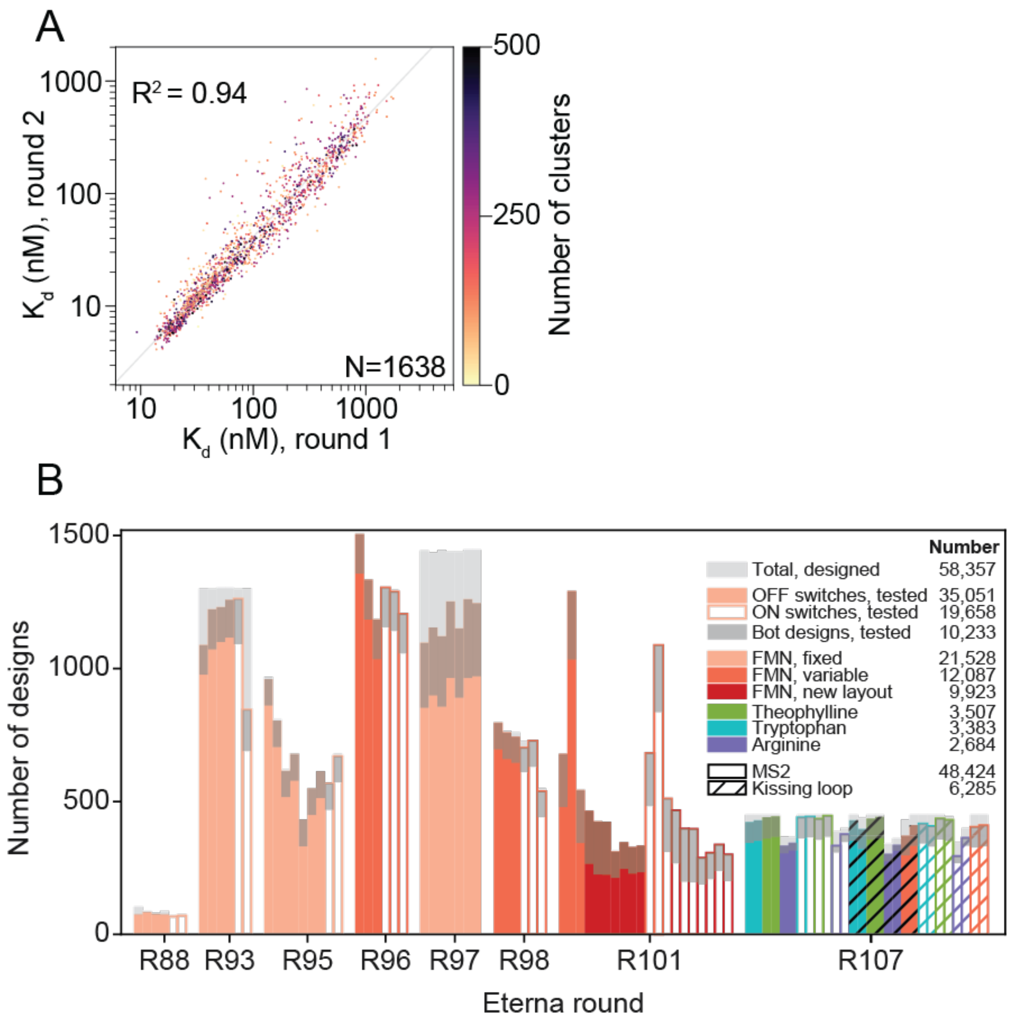

Fig. S1. Reproducibility and scale of RNA-MaP experiments. A) Reproducibility of a set of standard sequences spanning the range of detection. The replicates differ by time, protein preparation, DNA synthesis, and experimentalist. B) Number of Eterna designs created and tested. In total, 58,357 RNA switches were designed by the Eterna community, and the majority of these were successfully tested on the RNA array over seven Eterna rounds. The switches could either unfold the MS2 hairpin and turn off MS2 protein binding upon addition of ligand (OFF switches) or turn it on (ON switches). In each round, a minority of solutions were generated by computer algorithms (Ribologic). Three puzzle layouts were used for FMN. In the first (FMN, fixed), the position of both the FMN aptamer and the MS2 hairpin were fixed. In the second (FMN, variable), the MS2 hairpin could be placed in any location not overlapping the required aptamer residues. In the third (FMN, new layout), the position and direction of the aptamer arms as well as the sequence length in between them were changed. In the last round (R107), puzzles with new aptamers (Theophylline, Tryptophan, and Arginine) were designed, using either the previous MS2 reporter or a new Kissing loop reporter system. Dataset: Eterna results, also available on the Eterna website (https://eternagame.org).

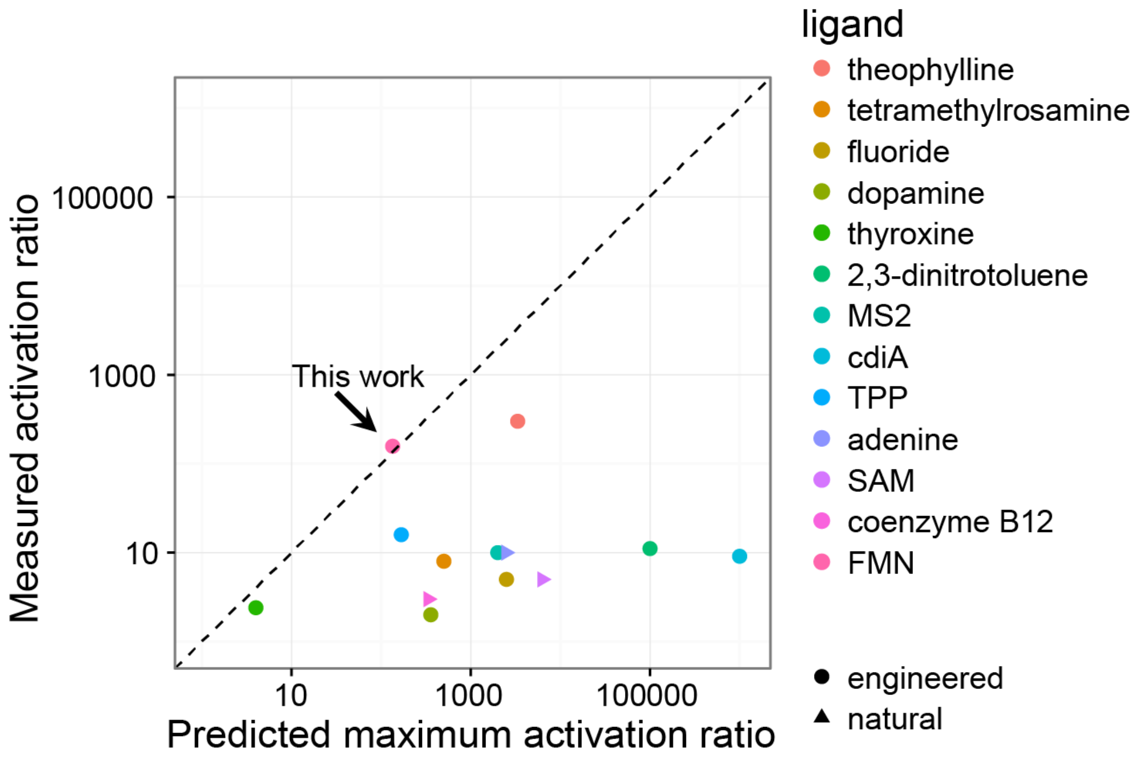

Fig. S2. Predicted and measured activation ratios for standalone RNA switches in the literature. Typically, the activation ratio (*AR*) is ~10-fold or below, less than the maximum predicted from the affinity of the ligand and the concentrations used in the experiments. The FMN/MS2 standalone RNA switches in this work (arrow) reach activation ratios nearly equal that of the maximally predicted value, calculated from the measured effective binding constants (K_D) and measured affinities for FMN (by chemical mapping) and MS2 (measured for controls on the RNA array in the same experiment).

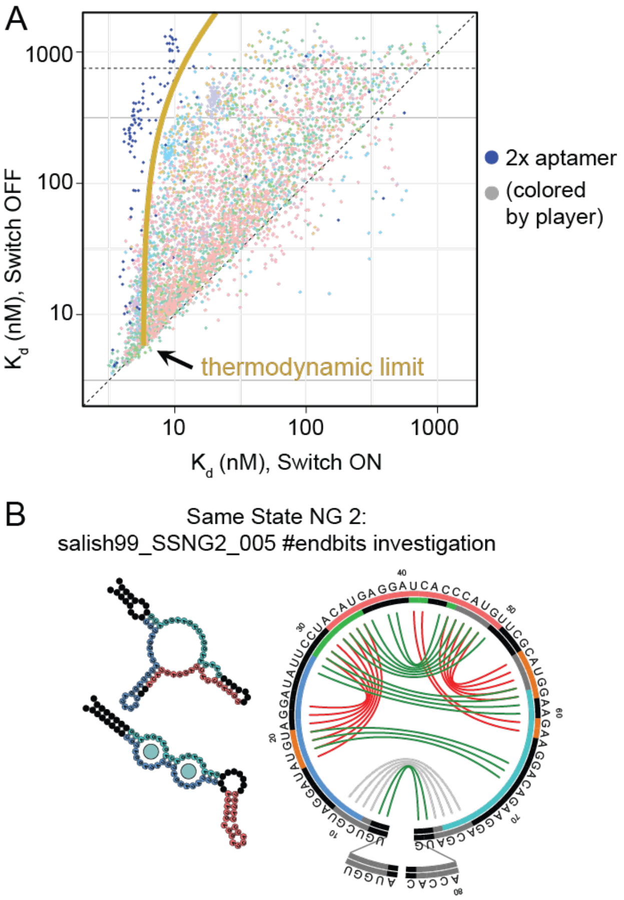

Fig. S3. **Cheat solutions with double input aptamers can surpass the thermodynamic limit for single input aptamers.** A) K_d values for both states of “Same State NG 2” puzzle designs. The predicted thermodynamic limit for the K_d in the OFF-state, based on the K_d in the ON-state, is shown in green. Player solutions (colored by player as in main text Fig. 2E) are shown, with solutions that use two FMN aptamer sequences colored in blue. These designs often exceed the calculated thermodynamic limit (gold curve). These “cheat” solutions exhibit high OFF-state K_d values while maintaining small K_ds in the ON-state. B) Predicted secondary structures for a well-performing double aptamer solution with predicted invariant base pairs (grey segments) and base pairs in the absence (red) or presence (green) of the FMN ligand. Outer circles display the MS2 hairpin and complementary segments in color; inner circle displays the FMN aptamer and complementary segments (as in main text Fig 2F).

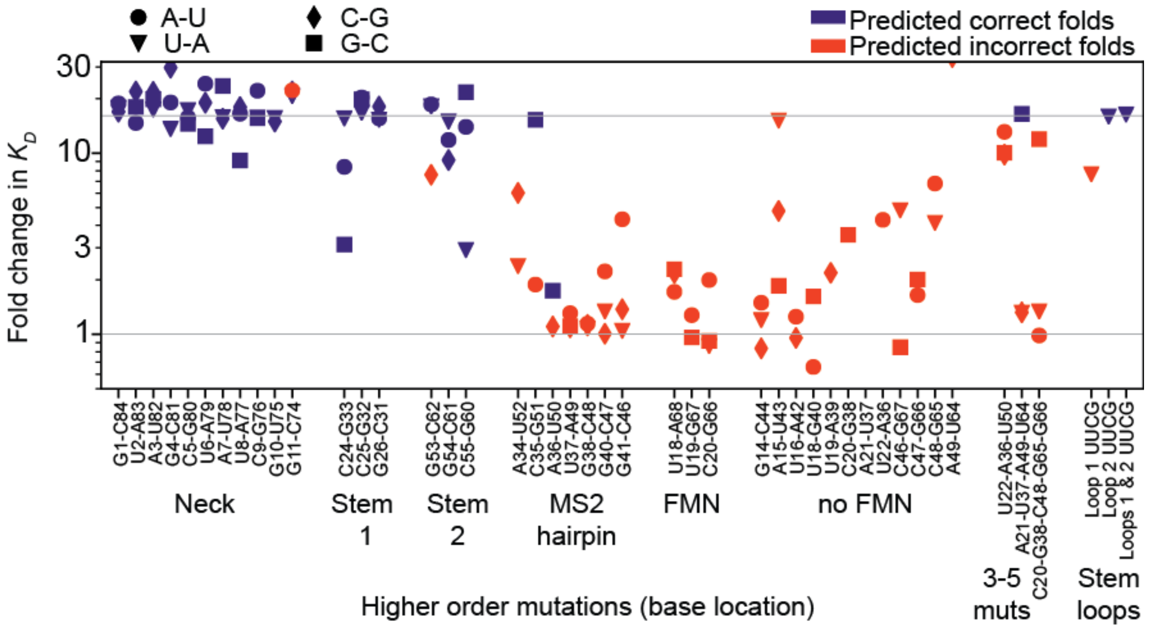

Fig. S4. Testing structural mechanisms by mutagenesis and functional measurements. Fold-change in K_d (activation ratio) for double and higher order mutants of the best Same State NG 2 puzzle solution (see table S2). Identical data as in Figure 2G but with all mutations indicated. All base pairs in at least one of the two secondary structures were mutated, changing the bases at that location to each of the other three canonical base pairs. Mutated sequences predicted to fold into the same secondary structures in the FMN and no FMN states are shown in blue. Most of these are located in the neck or the preserved stems. The other mutations, including those of the MS2 hairpin and the base pairs flanking the FMN aptamer, are predicted to break at least one of the two structures and are plotted in red. On the right are sequences with three or more base changes that preserve base pairing by propagating mutations or by mutating the stem tetraloops.

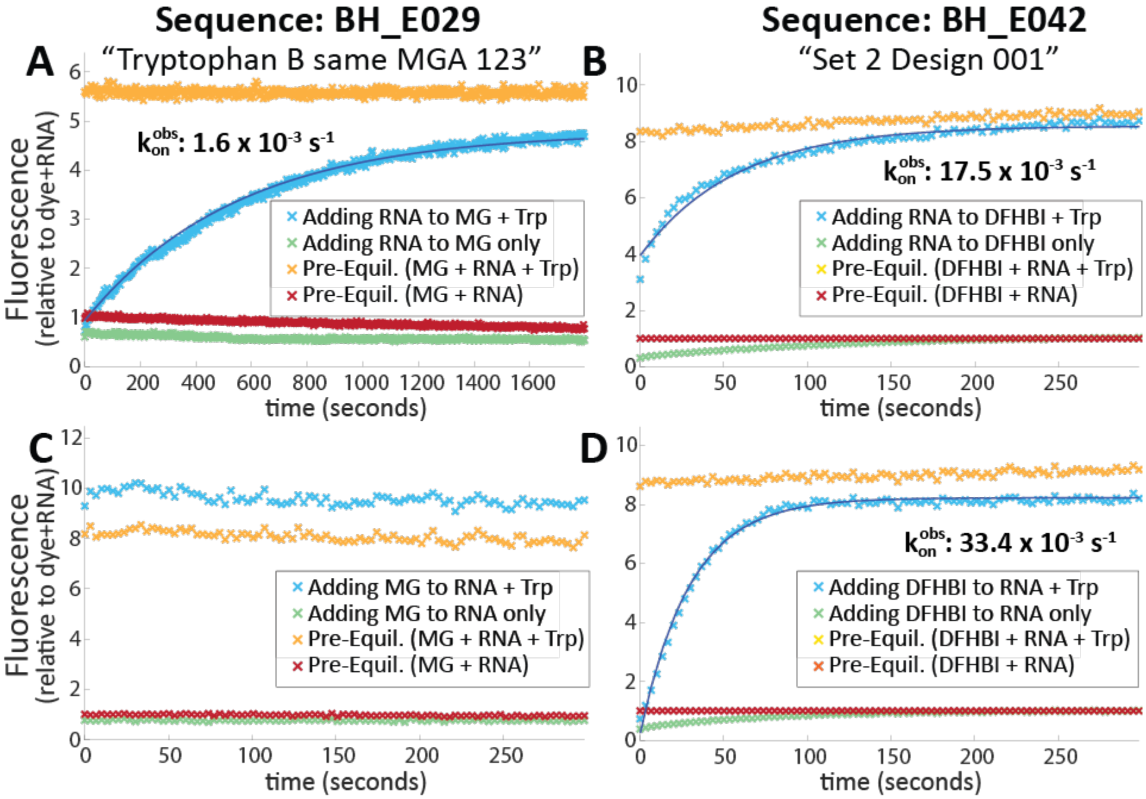

Fig. S5. Testing kinetics of ligand-triggered switching. Adding RNA into a solution of pre-equilibrated MG (A) or DFHBI (B) containing tryptophan (Trp) leads to variable rates of increasing signal (blue curves) approaching that of the same solution pre-mixed 30 minutes earlier (orange curves). The variable Trp association rates between these sensors—which contain identical Trp-binding aptamer motifs—suggests that structural rearrangement is the major energetic barrier for design BH_E029. In contrast, injecting MG (C) or DFHBI (D) into a solution of *pre-equilibrated* RNA + Trp (blue curve), we see that binding of the dye molecule happens rapidly, quickly approaching (or surpassing) the fluorescence signal of the same sample pre-mixed 30 minutes earlier (yellow curve). These data demonstrate switching kinetics on the order of 10s of seconds (for BH_E042) or minutes (BH_E029) with the slower switch likely gated by timescales of structural rearrangement. In all experiments, little fluorescence is detected for the analogous assay without any tryptophan (red and green curves), as expected for Trp-triggered light-up sensors.

	Puzzle name	Switch Design Round (Eterna Round)						
		1<br>R88	2<br>R93	3<br>R95	4<br>R96	5<br>R97	6<br>R98	7<br>R101
	**OFF switches**							
1.	Exclusion 1	75/104	1089/1301	960/968		1097/1443		
2.	Exclusion 2	85/87	1223/1300	804/809		1154/1437		
3.	Exclusion 3	77/81	1231/1303	615/620		1121/1445		
4.	Exclusion 4	75/88	1258/1303	678/680		1252/1439		
5.	Exclusion 5			432/432		1151/1439		
6.	Exclusion 6			549/549		1261/1446		
7.	Brourd's mod of Exclusion 4			613/613		1245/1446		
8.	Exclusion NG 1				1505/1518		796/797	679/679
9.	Exclusion NG 2				1333/1336		759/765	1291/1291
10.	Exclusion NG 3				1185/1186		743/759	543/544
11.	Inverted Exclusion NG 1							465/465
12.	Inverted Exclusion NG 2							425/426
13.	Inverted Exclusion NG 3							424/424
14.	Small Loop Exclusion NG 1							312/315
15.	Small Loop Exclusion NG 3							346/346
16.	Inverted Small Loop Exclusion NG 1							328/328
17.	Inverted Small Loop Exclusion NG 3							334/334
	**ON switches**							
18.	Same State 1	67/67	1262/1303	568/568				
19.	Same State 2	70/75	845/1302	670/679				
20.	Same State NG 1				1304/1305		702/726	682/682
21.	Same State NG 2				1289/1296		728/732	1088/1091
22.	Same State NG 3				1205/1207		538/550	511/511
23.	Inverted Same State NG 1							466/466
24.	Inverted Same State NG 2							398/398
25.	Inverted Same State NG 3							397/397
26.	Small Loop Same State NG 1							288/288
27.	Small Loop Same State NG 3							307/307
28.	Inverted Small Loop Same State NG 1							338/338
29.	Inverted Small Loop Same State NG 1							301/301

Table S1. Number of FMN/MS2 standalone RNA switches sequences designed and successfully tested per puzzle and round. Dataset: Eterna results, also available on the Eterna website (https://eternagame.org).

Puzzle Name (R107)	Max participant AR	Max Ribologic AR	Thermodynamic maximum AR
**Theo Exc A**	27	3.9	92
**Theo Exc B**	29	9.92	92
**Trp Exc A**	40.32	4.29	198
**Trp Exc B**	15.31	3.2	198
**Theo SS A**	13.83	2.6	92
**Theo SS B**	34.97	15.37	92
**Trp SS A**	13.95	4.55	198
**Trp SS B**	24.60	2.96	198

Table S2. Top activation ratios for MS2-activating sensors from Eterna players and Ribologic automated design, compared to thermodynamic maximum expected based on ligand concentrations. Data are from challenges without multiple rounds of refinement. Abbreviations: Theo, theophylline; Trp, tryptophan; Exc, exclusion (OFF-switches, ligand binding disfavors MS2 binding); SS, same-state (ON-switches, ligand binding favors MS2 binding); AR, activation ratio. Dataset: Eterna results, also available on the Eterna website (https://eternagame.org).

Aptamer	Conditions*	Mod	K_d	K_d error (+)	K_d error (-)	Units	Assay Conc.
FMN	RNA-MaP	1M7	1.71	0.60	0.44	µM	200 µM
Tetracycline	RNA-MaP	1M7	622.07	132.12	105.63	nM	60 µM
Tryptophan	RNA-MaP	DMS	12.09	10.50	5.19	µM	2.4 mM
Theophylline	RNA-MaP	DMS	13.11	9.21	4.86	µM	1.2 mM
FMN	Light-up	DMS	1.57	0.58	0.41	µM	200 µM
Tetracycline	Light-up	DMS	57.50	22.58	15.88	nM	60 µM
Tryptophan	Light-up	DMS	11.59	9.49	5.00	µM	2.4 mM
Theophylline	Light-up	DMS	239.54	161.15	96.27	µM	1.2 mM

* RNA-MaP solution conditions (100 mM Tris-HCl, pH 7.5, 80 mM KCl, 4 mM $MgCl_2$, 37 °C) and light-up sensor conditions (1x PBS, pH 6.1 (Gibson); 5 mM $MgCl_2$, 24 °C).

Table S3. Affinity constants as determined using dimethyl sulfate (DMS) or 1-methyl-7-nitroisatoic anhydride (1M7) chemical mapping.

Switch Design Round (Eterna Round)	Web page	Title
1 (R88)	https://eternagame.org/labs/4736274	MS2 Riboswitches On Chip
2 (R93)	https://eternagame.org/labs/5448678	MS2 Riboswitches On Chip - Round 2
3 (R95)	https://eternagame.org/labs/5807498<br>https://eternagame.org/labs/5736177	MS2 Riboswitches On Chip - Round 3<br>MS2 Riboswitches On Chip - Round 3 part 2
4 (R96)	https://eternagame.org/labs/5851792	Next Generation Riboswitches On Chip
5 (R97)	https://eternagame.org/labs/5962968	MS2 Riboswitches On Chip - Round 4
6 (R98)	https://eternagame.org/labs/6089624	Next Generation Riboswitches On Chip - Round 2
7 (R101)	https://eternagame.org/labs/6369201	FMN/MS2 Riboswitch Structure: the Paper
8 (R107)	https://eternagame.org/labs/7559749	Single-input switches, revisited

Table S4. Web links and titles for the Eterna labs in the study. The puzzles are available for design/game play and further information about the results are presented to Eterna participants.

1. J. Lee, W. Kladwang, M. Lee, D. Cantu, M. Azizyan, H. Kim, A. Limpaecher, S. Yoon, A. Treuille, R. Das, RNA design rules from a massive open laboratory. *Proc. Natl. Acad. Sci. U. S. A.* (2014), doi:10.1073/pnas.1313039111.
2. E. Osada, Y. Shimizu, B. K. Akbar, T. Kanamori, T. Ueda, Epitope mapping using ribosome display in a reconstituted cell-free protein synthesis system. *J. Biochem.* (2009), doi:10.1093/jb/mvp027.
3. R. Lorenz, S. H. Bernhart, C. Höner zu Siederdissen, H. Tafer, C. Flamm, P. F. Stadler, I. L. Hofacker, ViennaRNA Package 2.0. *Algorithms Mol. Biol.* (2011), doi:10.1186/1748-7188-6-26.

4. J. N. Zadeh, C. D. Steenberg, J. S. Bois, B. R. Wolfe, M. B. Pierce, A. R. Khan, R. M. Dirks, N. A. Pierce, NUPACK: Analysis and design of nucleic acid systems. *J. Comput. Chem.* (2011), doi:10.1002/jcc.21596.
5. S. K. Denny, N. Bisaria, J. D. Yesselman, R. Das, D. Herschlag, W. J. Greenleaf, High-Throughput Investigation of Diverse Junction Elements in RNA Tertiary Folding. *Cell* (2018), doi:10.1016/j.cell.2018.05.038.
6. R. Williams, S. G. Peisajovich, O. J. Miller, S. Magdassi, D. S. Tawfik, A. D. Griffiths, Amplification of complex gene libraries by emulsion PCR. *Nat. Methods* (2006), doi:10.1038/nmeth896.
7. I. Jarmoskaite, S. K. Denny, P. P. Vaidyanathan, W. R. Becker, J. O. L. Andreasson, C. J. Layton, K. Kappel, V. Shivashankar, R. Sreenivasan, R. Das, W. J. Greenleaf, D. Herschlag, A Quantitative and Predictive Model for RNA Binding by Human Pumilio Proteins. *Mol. Cell*. **74** (2019), doi:10.1016/j.molcel.2019.04.012.
8. R. She, A. K. Chakravarty, C. J. Layton, L. M. Chircus, J. O. L. Andreasson, N. Damaraju, P. L. McMahon, J. D. Buenrostro, D. F. Jarosz, W. J. Greenleaf, Comprehensive and quantitative mapping of RNA-protein interactions across a transcribed eukaryotic genome. *Proc. Natl. Acad. Sci. U. S. A.* (2017), doi:10.1073/pnas.1618370114.
9. M. J. Wu, J. O. L. Andreasson, W. Kladwang, W. Greenleaf, R. Das, Automated Design of Diverse Stand-Alone Riboswitches. *ACS Synth. Biol.* (2019), doi:10.1021/acssynbio.9b00142.
10. H. Wayment-Steele, M. Wu, M. Gotrik, R. Das, in *Methods in Enzymology* (2019).
11. S. Tian, J. D. Yesselman, P. Cordero, R. Das, Primerize: Automated primer assembly for transcribing non-coding RNA domains. *Nucleic Acids Res.* (2015), doi:10.1093/nar/gkv538.
12. S. Yoon, J. Kim, J. Hum, H. Kim, S. Park, W. Kladwang, R. Das, HiTRACE: High-throughput robust analysis for capillary electrophoresis. *Bioinformatics* (2011), doi:10.1093/bioinformatics/btr277.
13. W. Kladwang, T. H. Mann, A. Becka, S. Tian, H. Kim, S. Yoon, R. Das, Standardization of RNA chemical mapping experiments. *Biochemistry* (2014), doi:10.1021/bi5003426.

Eterna participants

Players from array rounds (186)

134543216789109876
200611736
a1b2
aditya1101
alacarus
Algebreaker
AndrewKae
Andrian Timeswift
Angus
anitamurray88
annakgonzalez
Anonymous
Ανώνυμος Αλκαλικός
Arnthi_470
Astromon
Atomos
averice
awesomehuman
beezurd
Beginner Chemist
ben_bell1
benajmin819
biggestlegoheroicafanever
Billy Reuben
biogirl.lake

BirchSci25
BlackLine02
Bloxanoid
bojan373
Brent Schofield
Brourd
c-quence
cake
casprus
cataway
ch.parushev@gmail.com
ChockfulaCola
chuckcoleman
cipheralex
clayishmantis
clollin
cmuevolveit
Control
csitron
cyborg
D'Wydd
darksearíver
dejerpha
DeNa
dick2014
dizzywings
dl2007
Dragonbard
DRdru
elaineng
Eli Fisker
ElNando888
Emily75
ererexiue
eternacac
Euphoria
fishfingure
FKJambalaya
garydfisher
GFRANK2
Giganox
gotauber
Gracier
Gres
HarryS
HMK
hoglahoo
holoaaron
hotcreek
hsl
Hyphema
Iaxtrdinar
IceBolt
James Collier
jandersonlee

janelle
janetmason
jayCampbell
JeNNeR
Jeshyr
jhartman
Jieux
jmf028
jnicol
jonah.pandey
JoshS5
JR
JSci
kawasakiqro
kchambliss
keepinggiant
kiriakos22
lamcho00
LarryB
LFP6
LloydHater
lluis
lrlee
lroppy
lukaszgorzel
macclark52
machinelves
maek666
Malcolm
Marzena11
mat747
mat747
Max Goff
Mayanne
Meechl
Megaterio_Rex
MeowNow360
missusweasley
Mr50Freemans
mrglenn
mspano
musicandscience
Mystem
nate_taylor
natybob
neo1096
Neoheurist
ngenuth
nkem
nohotbad
novice
oli.veri
Omei
oolong
Ozzy1313

PeAlc
Pi
pko
pmlkjn
pmreis
Poll na gColm
potterland
pwkr
quantropy
Raumlord
Remmy
rezaefar
rguioguio
RNAPRO2006
RunDMC
rxmullin
salish99
SaraBot
sbarrett125
sby1621
sdingle1
Sean O'Neil
sellena
sh'dynasty73
SigSig
skyblue
Snowpup
spvincent
Squeaky
starryjess
Stormseeker
student_5
Stump Cutter
Sunet
syang
Tatyana Zabanova
Tesla'sDisciple
TheDomBom13
Tigerlrg245
TlTIN
Trentis1
Tusaki
ViennaUCT
voyager1
wateronthemoon
wbt
whbob
Xtech
yuana
yunxiao
Zanna

Players within error of thermodynamic optimum for FMN switches:
Eli Fisker
jandersonlee

mat747
salish99
SaraBot

Lightning round players (solutions selected and synthesized): (19)
AndrewKae
Astromon
Brourd
cynwulf28
DigitalEmbrace
dl2007
Eli Fisker
Gerry Smith
jandersonlee
Jieux
JR
Malcolm
Omei
Poll na gColm
Ribologic
spvincent
TeslasDisciple
Zama
Zanna
